# Antibiotic Resistance of *Salmonella* Typhimurium Monophasic Variant 1,4,[5],12:i:-in China: A Systematic Review and Meta-Analysis

**DOI:** 10.3390/antibiotics11040532

**Published:** 2022-04-16

**Authors:** Xiaojie Qin, Mingzhe Yang, Hua Cai, Yangtai Liu, Leon Gorris, Muhammad Zohaib Aslam, Kai Jia, Tianmei Sun, Xiang Wang, Qingli Dong

**Affiliations:** 1 School of Health Science and Engineering, University of Shanghai for Science and Technology, Shanghai 200093, China; qxj19900709@163.com (X.Q.); 202572515@st.usst.edu.cn (M.Y.); usstlyt@163.com (Y.L.); zohaib.aslam.000@gmail.com (M.Z.A.); 201570147@st.usst.edu.cn (K.J.); 191690137@st.usst.edu.cn (T.S.); xiang.wang@usst.edu.cn (X.W.); 2 Shanghai Municipal Center for Disease Control and Prevention, Shanghai 200336, China; caihua@scdc.sh.cn; 3 Food Safety Futures, 6524 BS Nijmegen, The Netherlands; leon@foodsafetyfutures.org

**Keywords:** *Salmonella enterica* serovar 1,4,[5],12:i:-, antibiotic resistance, multidrug resistance, meta-analysis

## Abstract

Antibiotic resistance in *Salmonella* is a global public health problem. *Salmonella enterica* serovar 1,4,[5],12:i:- (*S*. 1,4,[5],12:i:-), a monophasic variant of *Salmonella* Typhmurium, is one of the leading *Salmonella* serovars in several countries. This study aimed to assess the prevalence of antibiotic resistance to this serovar in China through a systematic review and meta-analysis. Nineteen eligible studies during 2011–2021 were included. A total of 4514 isolates from humans, animals, foods, and the environment were reported, which mainly concerned isolates found in Guangdong, Guangxi, Jiangsu, and Shanghai. A random-effects model was used to estimate the pooled resistance rate of *S*. 1,4,[5],12:i:-. Rates were found to be very high (values ≥ 75%) for tetracycline, ampicillin, sulfisoxazole, and streptomycin; high (50–75%) for nalidixic acid, amoxicillin–clavulanic acid, and chloramphenicol; and moderate (25–50%) for trimethoprim–sulfamethoxazole, kanamycin, trimethoprim, and gentamicin. The rates of resistance to ciprofloxacin, cefotaxime, ceftriaxone, cefepime, ceftazidime, and colistin were low (values ≤ 25%), but of great concern in terms of their current clinical importance. Furthermore, a high multidrug resistance rate (86%, 95% CI: 78–92%) was present in *S*. 1,4,[5],12:i:-, with the ASSuT pattern largely dominating. Subgroup analysis results showed that the high heterogeneity of resistance rates was not entirely dependent on isolated sources. Taken together, the severity of antibiotic resistance in *S*. 1,4,[5],12:i:- urgently requires the rational use of antibiotics in future infection control and antibiotic stewardship programs.

## 1. Introduction

*Salmonella* is an important foodborne pathogen that has been estimated to cause 115 million human infections and 370,000 deaths per year globally [1]. *Salmonella* has been classified into 2659 serovars based on the unique combination of their surface antigens [2]. Among them, *Salmonella enterica* serovar Enteritidis and serovar Typhimurium are the most common causative agents of human non-typhoidal salmonellosis [3,4]. Notably, *Salmonella enterica* serovar 1,4,[5],12:i:- (*S*. 1,4,[5],12:i:-) is a monophasic variant of *Salmonella* Typhimurium that lacks the *fljB*-encoded second-phase H antigen. Its rapid spread across the world has notably affected human health in the last two decades [5,6,7,8,9]. It has been reported to be predominant in *S*. 1,4,[5],12:i:- isolates of various origins, for instance in USA, Italy, and Spain [10,11,12].

Since having been confirmed as an emerging pathogen, this particular serovar is now considered one of the most frequent *Salmonella* serotypes responsible for human infections. It has been recognized as causing human salmonellosis, particularly in countries and regions that are associated with the prominence of the swine-related food production chain [8,13,14,15,16,17]. Several foodborne outbreaks around the world caused by this monophasic serovar have since been reported due to contamination of pigs, and consequently pork and dried pork sausage products [5,15,16,17,18]. In Europe, it is the most common serovar among human, pig, and pork isolates [19,20,21], while in the United States it is in the top five serovars associated with human salmonellosis [15,22]. The serovar was ranked as the fourth most frequent serovar responsible for human salmonellosis in Guangdong province, China, since 2009 [23], and was found to be the fifth most frequent epidemic serovar in retail meat and meat production from 2011 to 2016 in China [16].

Next to the prevalence of *S*. 1,4,[5],12:i:-, its resistance to particular antibiotics [24,25,26,27,28] and its multidrug resistance profile [11,29,30,31] have been reported in several countries and regions globally. The majority of its isolates showed three prominent lineages at different times and in different regions. One of its clonal lines, identified as the “Spanish clone”, was specifically associated with pigs and pork products and phenotypically showed the multidrug resistance pattern ACSuGSTTm (i.e., ampicillin, chloramphenicol, sulfonamides, gentamicin, streptomycin, tetracycline, and trimethoprim) [32]. A clonal line referred to as the “USA clone” was associated with pigs. Notably, multidrug resistance of *S*. 1,4,[5],12:i:- strains was rare in the USA clones, which were different from Spanish clone isolates [12]. At present, the most prominent clonal line, designated as the “European clone” prevalence in pigs, has spread in several countries across the EU, for which ampicillin, streptomycin, sulfonamides, and tetracyclines (ASSuT) is the more prominent pattern [33]. In several provinces of China, such as Guangdong, Guangxi, and Jiangsu, the drug resistance phenotype of this serovar has been explored and it has been shown to be multidrug-resistant [34,35,36].

Concerning the genetic makeup of *S*. 1,4,[5],12:i:-, the genes underlying the ASSuT phenotype are present on a chromosomal resistance island that frequently includes *bla*_TEM_, *strA-strB*, *sul2*, and *tetB* [33,37]. The fluoroquinolone resistance-conferring genes *qnrS1* and *aac(6′)lb-cr* have been detected in MDR isolate *S*. 1,4,[5],12:i:- in Australia [38]. Extended-spectrum *β*-lactamase-encoding resistance genes (i.e., *bla*_CTX-M-1_, *bla*_CTX-M-14_) in *S*. 1,4,[5],12:i:- have been reported for isolates found in Germany and China [39,40], whereas plasmid-mediated colistin resistance genes, i.e., *mcr-1* and *mcr-3*, have been reported for isolates found in Switzerland, Australia, and China [38,41,42]. The aspects of the prevalence of antibiotic-resistant phenotypes in isolates from different sources, levels of antibiotic resistance, or MDR profiles in isolates *S*. 1,4,[5],12:i:- have been individually studied and documented in many different countries; however, there is a paucity in the literature of combinations of the various aspects mentioned in the context of China.

Therefore, this study aimed to quantify the prevalence of antibiotic resistance to *S*. 1,4,[5],12:i:- from humans, animals, foods, and environment sources in China based on relevant peer-reviewed publications available in the public domain, using the meta-analysis approach. A better understanding of antibiotic resistance prevalence and rates for individual antibiotic agents, as well as of MDR features, was sought to provide concrete information for relevant risk managers and policymakers deciding on preventive and therapeutic human clinical strategies or regulatory measures concerning food and food production regarding public health safety, including the utility of antibiotics in the food chain.

## 2. Results

### 2.1. Characteristics of Eligible Studies and Datasets

**Figure 1:**
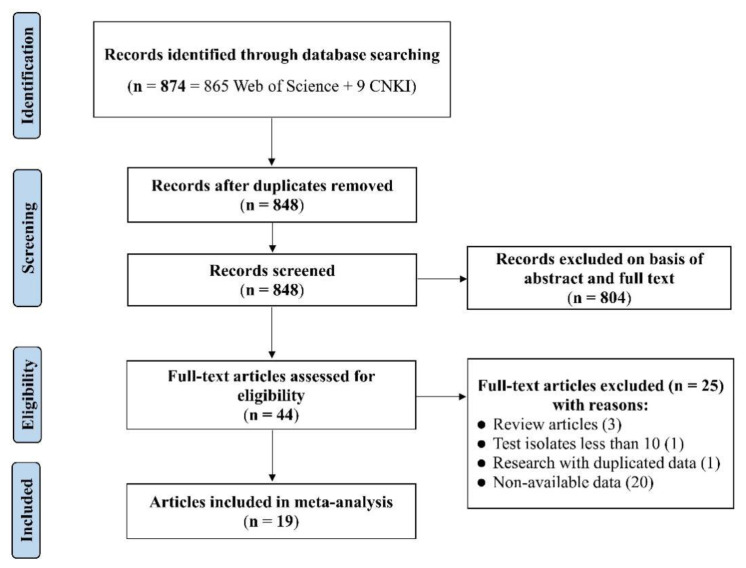
PRISMA flowchart diagram for the selection process of eligible studies.

The detailed flowchart of the literature meta-analysis is shown in Figure 1. A total of 874 potentially relevant *S*. 1,4,[5],12:i:- publications were identified in the online databases considered based on the keywords used, from which 26 duplicated articles were removed. The remaining 848 articles were screened for relevant information on antibiotic resistance, which removed 804 articles. The remaining 44 articles were evaluated against the pre-established eligibility criteria. As a result, 25 articles were excluded due to low numbers of isolates (<10), reporting duplicated data, or non-availability data on resistance rates in the literature. Finally, 19 articles (i.e., 12 in English and 7 in Chinese) were included in the outcome of the meta-analysis, reporting on antibiotic resistance of *S*. 1,4,[5],12:i:- isolates from different sources in China in the period 2011–2021 (Table S1). Collectively, they are referred to as eligible studies. It should be noted that these eligible studies may not represent the insights into the antibiotic resistance traits of isolates of this serovar across all of China that a systematic and targeted investigation would have achieved, but rather provide an indication of the situation and the potential relevance for more targeted investigations and surveillance.

**Figure 2:**
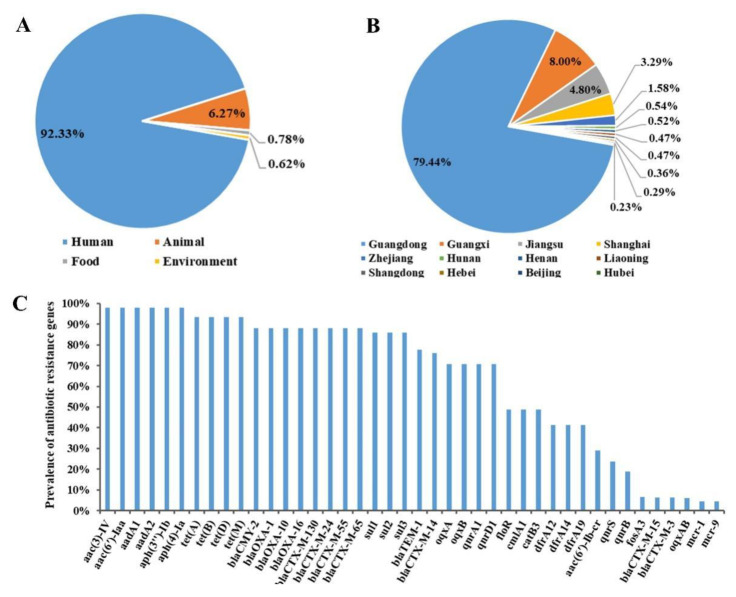
Data on isolates of S. 1,4,[5],12:i:- extracted from eligible studies concerning (**A**) sources, (**B**) geographical locations in China, and (**C**) detection rates of antibiotic resistance genes.

Following full-text quality checking, 186 sets of antibiotic resistance and MDR data for *S*. 1,4,[5],12:i:- were retrieved from the 19 eligible reports, encompassing a total of 4514 isolates derived from humans, animals, foods, and environment sources. Human-derived isolates accounted for the highest proportion retrieved from the eligible studies, followed by animal-derived isolates, then smaller numbers of isolates from foods and environment sources (Figure 2A). Of these, notably 74% of the human-derived isolates were from infants younger than 3 years old, while 98% of the animal-derived isolates were from pigs; food-derived isolates were mainly from pork and beef products, whereas all environment-derived isolates were from a single pig farm. As for the geographic origin of the isolates reported in eligible studies, these were mainly from provinces and municipalities in the eastern part of China, such as Guangdong, Guangxi, Jiangsu, Shanghai, Zhejiang, Liaoning, Shandong, Hunan, Henan, Beijing, Hebei, and Hubei (Figure 2B). Among them, by far the highest proportion of isolates was from Guangdong, followed by Guangxi, Jiangsu, and Shanghai.

Information concerning antibiotic resistance genes of the serovar was included in four eligible studies (three in English and one in Chinese). The rate of occurrence of antibiotic resistance genes was determined by dividing the number of resistant *S*. 1,4,[5],12:i:- isolates by the number of total *S*. 1,4,[5],12:i:- isolates, as presented in Figure 2C. A total of 42 genes conferring resistance against antibiotics were identified for *S*. 1,4,[5],12:i:- isolates reported in the eligible studies. The most prominent resistance genes were those conferring resistance against aminoglycosides (*aac(3)-IV*, *aac(6′)-Iaa*, *aadA1*, and *aadA2*), tetracyclines (*tetA*, *tetB*, *tetD*, and *tetM*), *β*-lactamases (*bla*_TEM_, *bla*_CTX-M_, *bla*_OXA_, and *bla*_CMY-2_), sulfonamides (*sul1*, *sul2*, *sul3*, *dfrA1*, and *dfrA12*), quinolones (*oqxAB*, *qnrB*, and *qnrS*), chloramphenicol (*cmlA*, *catB*, and *floR*), and colistin (*mcr-1*, and *mcr-9*).

### 2.2. Pooled Prevalence of Antibiotic Resistance in S. 1,4,[5],12:i:-

In all eligible studies, the antibiotic susceptibility of *S*. 1,4,[5],12:i:- was interpreted according to Clinical and Laboratory Standards Institute CLSI guidelines. Data on seven major types of antibiotics, including 17 individual antibiotic agents, were covered in the studies resulting from the meta-analysis, allowing us to calculate antibiotic resistance rates for *β*-lactams (i.e., ampicillin, amoxicillin–clavulanic acid, cefotaxime, ceftazidime, cefepime, and ceftriaxone), aminoglycosides (i.e., gentamicin, streptomycin, and kanamycin), sulfonamides (i.e., trimethoprim–sulfamethoxazole, sulfisoxazole, and trimethoprim), phenicols (i.e., chloramphenicol), polymyxin (i.e., colistin), quinolones (i.e., ciprofloxacin and nalidixic acid), and tetracyclines (i.e., tetracycline). Eight of these antibiotic agents (i.e., ampicillin, ceftazidime, gentamicin, streptomycin, trimethoprim–sulfamethoxazole, chloramphenicol, ciprofloxacin, and tetracycline) were reported in at least 70% of the eligible studies.

The meta-analysis results on the prevalence and heterogeneity of antibiotic resistance in *S*. 1,4,[5],12:i:- are shown in Table 1. Based on an arbitrary categorization of resistance rates into very high (≥75%), high (50–75%), moderate (25–50%), and low (≤25%), resistance rates of *S*. 1,4,[5],12:i:- isolates to different antibiotic agents were found to vary from very high to low as follows (with their 95% confidence interval in parentheses): tetracycline, 93% (90–95%); ampicillin, 91% (83–94%); sulfisoxazole, 86% (76–93%); streptomycin, 76% (66–83%); nalidixic acid, 73% (69–76%); amoxicillin–clavulanic acid, 70% (26–94%); chloramphenicol, 54% (47–60%); trimethoprim–sulfamethoxazole, 48% (33–63%); kanamycin, 45% (40–50%); trimethoprim, 45% (39–52%); gentamicin, 44% (29–59%); ciprofloxacin, 22% (15–32%); ceftriaxone, 21% (10–38%); cefotaxime, 19% (12–29%); cefepime, 12% (9–15%); ceftazidime, 10% (7–15%); colistin, 2% (1–5%).

A significant low heterogeneity was observed for tetracycline (46%; *p* < 0.1) among the eligible studies (Table 1), indicating a relatively low variance in data for this antibiotic agent. Rather high levels of heterogeneity and wide CI ranges for resistance rates derived from the literature were observed for the other antibiotic agents. For instance, as shown in Table 1, heterogeneities were significant (*p* < 0.1) and high for amoxicillin–clavulanic acid (96%), gentamicin (91%), ciprofloxacin (90%), cefotaxime (89%,), chloramphenicol (88%), trimethoprim–sulfamethoxazole (86%), sulfisoxazole (83%), trimethoprim (83%), streptomycin (79%), and ceftazidime (74%). A significant moderate level was derived for ampicillin (63%). There were lower, non-significant heterogeneity values regarding the antibiotic resistance of *S*. 1,4,[5],12:i:- to cefepime, ceftriaxone, kanamycin, and nalidixic acid, which may be due to the small number of relevant studies.

### 2.3. Pooled Prevalence of Multiple-Drug Resistance in S. 1,4,[5],12:i:-

The results of the meta-analysis on the prevalence and heterogeneity of *S*. 1,4,[5],12:i:- MDR are presented in Table 2. The prevalence of MDR in isolates of the serovar was 86% (95% CI: 78–92%) out of 2251 isolates for which resistance was reported, indicating that the majority of the *S*. 1,4,[5],12:i:- strains showed resistance to three or more antibiotics. Notably, the prevalence rates in *S*. 1,4,[5],12:i:- isolates of ASSuT, ACSSuT, and ACSuGSTTm MDR profiles were 70% (95% CI: 66–73%), 29% (95% CI: 18–43%), and 27% (95% CI: 24–31%), respectively. The heterogeneity in data across the eligible literature was high at a significant level.

### 2.4. Prevalence of Antibiotic-Resistant S. 1,4,[5],12:i:- from Different Isolated Sources

**Figure 3:**
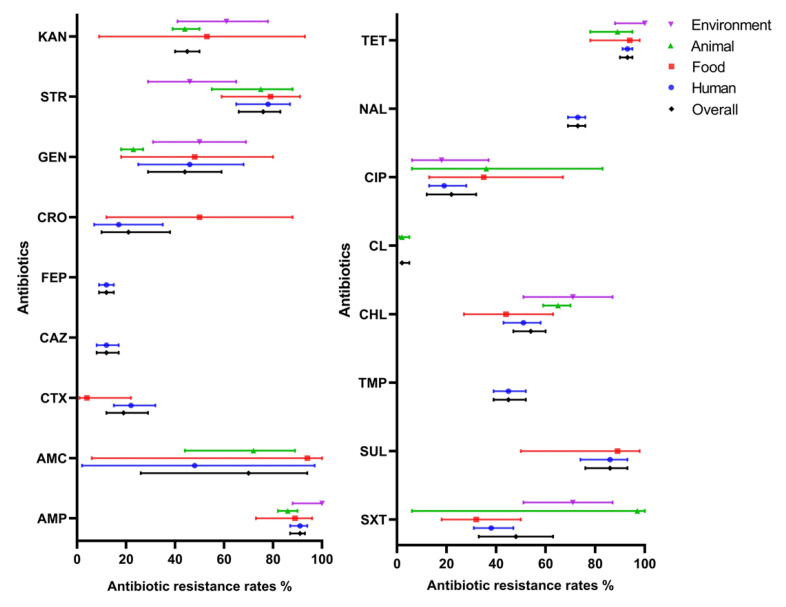
Resistance rate of *S*. 1,4,[5],12:i:- to antibiotics by sampling sources. Antibiotics in the left picture: AMP, ampicillin; AMC, amoxicillin–clavulanic acid; CTX, cefotaxime; CAZ, ceftazidime; FEP, cefepime; CRO, ceftriaxone; GEN, gentamicin; STR, streptomycin; KAN, kanamycin. Antibiotics in the right picture: SXT, trimethoprim–sulfamethoxazole; SUL, sulfisoxazole; TMP, trimethoprim; CHL, chloramphenicol; CL, colistin; CIP, ciprofloxacin; NAL, nalidixic acid; TET, tetracycline. Lines indicate 95% confidence intervals.

Given that there was considerable heterogeneity among the eligible studies retrieved and with an aim to analyze possible reasons for this, stratified analyses were used to assess heterogeneity across subgroups defined by the various source that isolates were obtained from humans, animals, foods, and the environment. The results are shown in Figure 3. In many cases, quite broad ranges of antibiotic resistance were deduced from the available data and there was substantial variation in occurrence across sources. For instance, some antibiotic agents were only reported from human sources (i.e., ceftazidime, cefepime, trimethoprim, and nalidixic acid), while others were not reported from animal sources (cefotaxime, ceftriaxone, and sulfisoxazole) or were reported from all sources (i.e., ampicillin, chloramphenicol, ciprofloxacin, gentamicin, streptomycin, tetracycline and trimethoprim–sulfamethoxazole). Of the agents reported from food and animal sources, relatively high resistance rates were observed in the case of ampicillin, streptomycin, sulfisoxazole, and tetracycline.

### 2.5. Prevalence of Multiple-Resistant S. 1,4,[5],12:i:- from Different Geographical Regions in China

**Figure 4:**
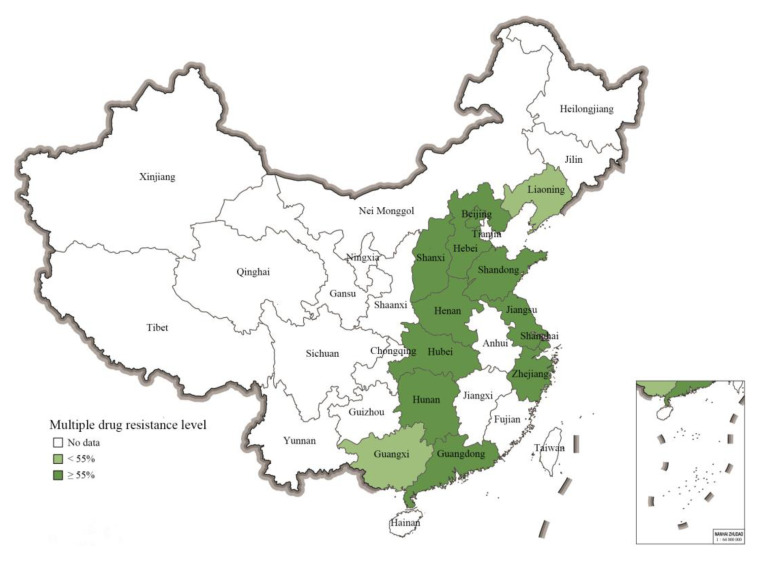
The pooled prevalence rates of MDR *S*. 1,4,[5],12:i:- in 13 Chinese provincial regions based on the eligible studies.

Albeit the limitations of a meta-analysis study, the MDR prevalence data on *S*. 1,4,[5],12:i:- covered an important number of provinces (*n* = 11) and municipalities (*n* = 2) in the eastern part of China. As shown in Figure 4, the mean pooled prevalence of MDR-level values for *S*. 1,4,[5],12:i:- isolates (calculated with a CI of 95% in all cases) in different regions were as follows: Shaanxi, 100% (40–100%); Hubei, 100%, (69–100%); Shandong, 96% (31–100%); Hunan, 96% (76–99%); Hebei, 94% (66–99%); Zhejiang, 93% (42–100%); Shanghai, 92% (71–98%); Jiangsu, 91% (86–94%); Henan, 91% (71–98%); Beijing, 85% (55–96%); Guangdong, 81% (73–87%); Guangxi, 54% (49–59%); Liaoning, 31% (14–57%). Notably, the actual numbers of isolates from different parts of China reported on in the eligible studies varied strongly. Consequently, the numbers of isolates displaying MDR also varied between provinces and municipalities, including Guangdong (*n* = 1340), Guangxi (*n* = 355), Hunan (*n* = 24), Henan (*n* = 23), Shandong (*n* = 21), Hebei (*n* = 16), Hubei (*n* = 10), and Shaanxi (*n* = 4).

## 3. Discussion

A meta-analysis approach was used to retrieve relevant published data concerning the prevalence and antibiotic resistance traits of isolates of *S*. 1,4,[5],12:i:- in China, considering that this serovar has become prevalent in Asia, Europe, Africa, North America, and South America and has been implicated in several outbreaks of salmonellosis [8,9,19,43]. It should be noted that the data retrieved by this meta-analysis study do not provide a complete and balanced picture of the *S*. 1,4,[5],12:i:- situation across China, such as would be obtained through systematic surveillance and targeted research. The study may rather reflect the interests of researchers in this particular serovar and their choices concerning the study scope, data generation, and region. Despite this caveat, the results of this study may still provide useful insights for government decisionmakers and others on the relevance and urgency to consider the impacts of the spread of *S*. 1,4,[5],12:i:- in China and the antibiotic resistance properties of isolates of this serovar.

Of the 4514 *S*. 1,4,[5],12:i:- isolates in China reported on in the studies retrieved from the literature, about 92% was from humans. Of these isolates from human sources, 74% were from infants and young children under 3 years old, most probably indicating that this specific serovar is predominantly prevalent in these hosts because of its high infection rate in them. High infections rate in infants and young children have been reported before [26,35]. Such a host infection rate may be quite universal. Concerning the reported prevalence of the serovar in non-human sources, about 6% of isolates were from animal sources, while the remainder was from food sources and a pig farm (less than 1% of the isolates in each case). Of the animal associated isolates, 98% was from pigs, which is in line with earlier publications noting pigs, pork, and pork products as the main vehicle for *S*. 1,4,[5],12:i:- [17,28,44,45]. A relatively high prevalence of the serovar in pig sources has also been reported for the USA (18.7%) and EU (48%) [19,46]. However, compared to these countries, the proportion of the serovar in non-human sources in China is relatively low, which may be due to the lack of consistent surveillance of and research on this serovar across the country, and the limitations of the detection method.

In terms of antibiotic resistance, this study identified very high or high levels of resistance of *S*. 1,4,[5],12:i:- isolates from various sources to tetracycline (93%), ampicillin (91%), sulfisoxazole (86%), streptomycin (76%), nalidixic acid (73%), amoxicillin–clavulanic acid (70%), and chloramphenicol (54%), which is consistent with previous results [36,47,48]. *S*. 1,4,[5],12:i:- resistance rates to other antibiotics (e.g., ceftriaxone, cefotaxime, kanamycin, and ciprofloxacin) in China were low to moderately high. Notably, high levels of heterogeneity were observed in the resistance rates calculated for most antibiotics, which may be related to the fact that the overwhelming amount of isolates in the eligible studies were from human sources, leaving a limited number of isolates from other sources in the pooled data for the resistance rate calculation. Nevertheless, high antibiotic resistance rates were noted for multiple key antibiotics, which may be the result of improper use or misuse of these antibiotics in human clinical treatment and animal food production. The risk is that improper use and misuse of antibiotics may select for antibiotic-resistant bacteria, since such uses can result in sublethal exposure of bacteria to antibiotics, possibly generating antibiotic-resistant bacteria. Inadequate antibiotic concentrations may occur in patients and livestock during antibiotic therapy, resulting in resistance bacteria that are widely distributed in the environment such as sewage water, rivers, lakes, and even drinking water [49]. Importantly, the increased antibiotic resistance levels of *S*. 1,4,[5],12:i:- isolates may contribute to further spread of the serovar, suggesting the importance of establishing targeted nation-wide surveillance to obtain a better view of the current spread of *S*. 1,4,[5],12:i:- and its prevalence in different sources, such that suitable risk mitigation measures can be established.

Although a limited number of different sources have been reported in the studies retrieved through the meta-analysis here, the occurrence of antibiotic-resistant *S*. 1,4,[5],12:i:- isolates in humans, animals, foods, and the environment suggests that resistant isolates of the serovar are probably widespread in China. Of the four antibiotic resistances noted in *S*. 1,4,[5],12:i:- isolates exclusively obtained from humans, rates against ceftazidime and cefepime were low (10 and 12%, respectively), while rates against nalidixic acid and trimethoprim were higher (73 and 45%, respectively). The latter antibiotics may have been used for longer or more intensively in human clinical treatment than the other antibiotic agents, leading to increased resistance rates. Extensive and imprudent usage of antimicrobials in veterinary practices and animal husbandry may lead to increased antibiotic resistance rates [50,51], and in this study high resistance rates were for instance found against ampicillin, chloramphenicol, and tetracycline, which are often used in animal treatments. In addition, differences in antibiotic resistance among isolates from different sources may be mainly related to the limited number of isolates from animals, foods, and the environment.

Previous studies have already shown that *S*. 1,4,[5],12:i:- isolates may display multiple drug resistance, which may be an important reason for their rapid spread since it emerged some decades ago [52,53]. Genes conferring resistance against quinolones, tetracyclines, aminoglycosides, *β*-lactam, and chloramphenicol are often located in transferable genetic elements, such as integrons and plasmids [54,55]. In the investigations retrieved in this study, numerous antibiotic-resistant genes located in chromosome and mobile genetic elements were detected in *S*. 1,4,[5],12:i:- isolates (i.e., *aac(3)-IV*, *aac(6′)-Iaa*, *bla*_TEM_, *bla*_CTX-M_, *sul*, *tet*, and *mcr*). Additionally, several plasmid-mediated quinolone resistance genes (i.e., *oqxAB*, *qnrS*, and *qnrB*) were also identified. The presence of the above-mentioned resistance genes is consistent with the antibiotic resistance phenotypes of the *S*. 1,4,[5],12:i:- isolates. Previous studies also reported these resistance genes in the serovar, next to others. For instance, genes encoding *β*-lactamase (*bla*_TEM_, *bla*_CTX-M_), aminoglycosides (*aac(3)-IV*, *aac(6′)-Iaa*, *aadA1*, and *aadA2*), tetracyclines (*tetA*, *tetB*), sulfonamides (*sul*, *dfrA1*, and *dfrA12*), quinolones (*oqxAB*, *qnrB*, and *qnrS*), chloramphenicol (*cmlA*, *catB*), and colistin (*mcr-1*, and *mcr-3*) have been observed in isolates from different sources in Germany [37], Spain [32], Portugal [56], Canada [29], and Thailand [17].

Given that MDR is a factor that may significantly contribute to the potential of *S*. 1,4,[5],12:i:- to spread in and across countries, it has been identified as a particular concern globally. In this study, 86% of 2251 *S*. 1,4,[5],12:i:- isolates were found to be multidrug-resistant strains. This rate is in the same ballpark as some MDR rates reported elsewhere, such as the 92% rate in *S*. 1,4,[5],12:i:- isolates from different origins and regions in Portugal [57], the 81.4% rate reported for human isolates in the EU [4], the 84.7% rate in swine isolates [36], and the 76.9% rate in food isolates [58]. For the *S*. 1,4,[5],12:i:- isolates from China involved in this study, three MDR patterns were noted, namely ASSuT, ACSSuT, and ACSuGSTTm, with the ASSuT pattern largely dominating in terms of prevalence (70%). In the EU this particular pattern also dominates [33], although isolates with an ACSuGSTTm pattern typically are associated with pigs and pork products in Spain [32]. The highest absolute numbers of isolates showing MDR were from Guangdong (*n* = 1340) and Guangxi (*n* = 355). The MDR rates determined for these provinces (mean values of 81% and 54%, respectively) may be more representative of the magnitude of the spread of multidrug-resistant isolates of *S*. 1,4,[5],12:i:- than the often higher prevalence rates derived for various other provinces and municipalities, for which only very limited numbers of isolates were available in the eligible studies retrieved. Guangdong and Guangxi are provinces with considerable levels of pig production. Considering the high prevalence of isolates of *S*. 1,4,[5],12:i:- in pigs and pork products and especially the potential exacerbating effect of multidrug-resistant strains, as well as the importance of pork production in China as a whole, it is of concern that systematic monitoring of this pathogen in pigs and pork products is lacking in China.

The isolates of *S*. 1,4,[5],12:i:- in this study were mainly from Eastern China according to the eligible studies. Except for Guangdong, Guangxi, Jiangsu, and Shanghai, the numbers of strains from other provinces are very limited, so the results of this study cannot fully reflect the overall drug resistance of *S*. 1,4,[5],12:i:- in China. Most of the isolates were derived from clinical patients, while the numbers of isolates from animals, foods, and the environment were relatively limited, so more surveillance data are needed to accurately reflect the antibiotic resistance of isolates from these sources.

## 4. Materials and Methods

### 4.1. Search Strategy

Two databases were systematically searched to collect potentially relevant publications, namely Web of Science and China National Knowledge Infrastructure (CNKI). The following keywords and their combinations were used in Web of Science: “*Salmonella enterica* Serotype 4,5,12:i:-” OR “*Salmonella* 4,5,12:i:-” OR “*Salmonella* 1, 4,[5],12:i:-” OR “*Salmonella* Typhimurium-like” OR “Monophasic *S*. Typhimurium” OR “Monophasic variants of *Salmonella* Typhimurium-like” OR “*S*. 4,12:i:-” OR “*S*. 4,5,12:i:-” OR “Monophasic *Salmonella*” OR “*Salmonella* 1,4,[5],12:i:-” OR “1,4,[5],12:i:-” OR “1,4,5,12:-:-” OR “1:4,[5],12:i:-”. Search terms (in Chinese) used for searching in CNKI were: “*Salmonella enterica* Serotype 4,5,12:i:-” OR “*Salmonella* 1,4,[5],12:i:-” OR “1,4,[5],12:i:-” OR “Monophasic *Salmonella* Typhimurium” OR “1,4,[5],12:i:- *Salmonella*” OR “*Salmonella* 4,[5],12:i:-” OR “*Salmonella* Typhimurium Monophasic variant” OR “*Salmonella* 1,4,[5],12:i:-”. Subsequently, the cited references of retrieved articles were manually searched to identify any additional relevant studies. Searches were performed between XJQ and MZY.

### 4.2. Selection Criteria

The Preferred Reporting Items for Systematic Reviews and Meta-Analyses (PRISMA) statement approach was employed for reporting the literature screening and retrieval process and outcomes [59]. After removing duplicate records, all publications were checked against the following set of selection criteria. A study was considered eligible if it contained information about specific antibiotic agent resistance or MDR patterns of isolates of *S.* 1,4,[5],12:i:-, as well as the percentage of specific antibiotic agent resistance or MDR of isolates. A study was excluded if (1) it was a duplicate report; (2) it was published as a conference abstract or as a review; (3) it was not relevant to antibiotic prevalence, resistance, or MDR levels, such as studies rather focusing on plasmids, comparative genomes, or virulence; or (4) the total number of isolates was lower than 10. In this study, only original research articles were included.

### 4.3. Data Extraction

The following specific data concerning various aspects of antibiotic resistance of *S*. 1,4,[5],12:i:- in China mentioned in articles retrieved on the basis of the selection criteria were extracted: author(s), publication year, sampling location(s), the total number of tested isolates, the total number of resistant isolates, antibiotic agents tested, antibiotic resistance levels, and MDR rates, the origin of isolates, method for drug susceptibility test, and antibiotic resistance gene information.

### 4.4. Meta-Analysis and Statistical Analysis

This meta-analysis was performed using R-software (meta-package, version 4.9.2; The University of Auckland). Rates of antibiotic resistance were estimated by the number of antibiotic resistance isolates divided by the total number of isolates expressed as percentages. A random-effects model was applied to analyze the pooled resistance rate of *S*. 1,4,[5],12:i:-. In all instances, rates were calculated using a 95% confidence interval (CI). Heterogeneity among studies was assessed using Cochran’s Q-test (significance level of *p* < 0.1) and the *I*^2^ statistic [60]. *I*^2^ values with percentages of 25%, 50%, and 75% were considered low, moderate, and high heterogeneity levels, respectively [61]. A subgroup analysis was carried out to explore the possible reasons for heterogeneity based on the source of isolates (i.e., humans, animals, foods, environment). All statistical tests with a Q-test value (*p*) < 0.05 were considered significant.

## 5. Conclusions

The data retrieved in this study indicate that *S*. 1,4,[5],12:i:- is already highly prevalent in some provinces in China, and it poses a significant risk to humans due to further spread of the serovar. Factors exacerbating the risk posed by the serovar are its resistance to antibiotics and its multidrug resistance properties. Resistance of *S*. 1,4,[5],12:i:- isolates to antibiotics was found to be reported as very high (i.e., tetracycline, ampicillin, sulfisoxazole, streptomycin) or high (i.e., nalidixic acid, amoxicillin–clavulanic acid, and chloramphenicol), while the resistance rate for clinically important antibiotics, such as ciprofloxacin and cefotaxime, is low at present according to this study. Furthermore, MDR may contribute to the rapid spread of the serovar, and a high level of MDR was evident in the *S*. 1,4,[5],12:i:- isolates from China, in which ASSuT was the most predominant MDR pattern and numerous antibiotic resistance genes were detected. More systematic, targeted surveillance and research into the prevalence rates of antibiotic resistance and MDR of *S*. 1,4,[5],12:i:- in humans, animals, foods, and the environment are needed to provide the necessary data on the levels of resistance to key antibiotics to inform management decisions related to the use of antibiotics. Future research should further explore the paths of the evolution and transmission of this relatively recently emerged serovar, particularly in food and food-producing environments, in order to better understand its occurrence and spread valuable information to mitigate its further spread in China and to address forthcoming emerging serotypes.

## Figures and Tables

**Table 1:** Meta-analysis results concerning antibiotic resistance in *S*. 1,4,[5],12:i:- isolates in China.

Antibiotics		Total Number of Isolates	Resistance Rate		Heterogeneity	
			Mean of Pooled Resistance Rate %	95% CI (%) ^1^	*I*^2^(%) ^2^	*Q-p* ^3^
*β*-Lactams	Ampicillin	4536	91	83–94	63	<0.1
	Amoxicillin–clavulanic acid	579	70	26–94	96	<0.1
	Cefotaxime	4151	19	12–29	89	<0.1
	Ceftazidime	4128	10	7–15	74	<0.1
	Cefepime	498	12	9–15	0	0.98
	Ceftriaxone	33	21	10–38	1	0.36
Aminoglycosides	Gentamicin	4520	44	29–59	91	<0.1
	Streptomycin	1701	76	66–83	79	<0.1
	Kanamycin	363	45	40–50	20	0.28
Sulfonamides	Trimethoprim–sulfamethoxazole	3179	48	33–63	86	<0.1
	Sulfisoxazole	1347	86	76–93	83	<0.1
	Trimethoprim	1340	45	39–52	83	<0.1
Phenicols	Chloramphenicol	4443	54	47–60	88	<0.1
Polymyxins	Colistin	255	2	1–5	—	—
Quinolones	Ciprofloxacin	4532	22	15–32	90	<0.1
	Nalidixic acid	498	73	69–76	0	0.35
Tetracyclines	Tetracycline	4447	93	90–95	46	<0.1

^1^ CI: Confidence interval. ^2^ *I*^2^: Index for the degree of heterogeneity. ^3^ Cochran’s Q-test *p* value.

**Table 2:** Meta-analysis results concerning MDR rates in *S*. 1,4,[5],12:i:- isolates in China.

Multiple	Total Number of Isolates	MDR Rate		Heterogeneity	
		Mean of Pooled MDR Rate %	95% CI (%) ^1^	*I*^2^(%) ^2^	*Q-p* ^3^
≥3	2251	86	78–92	87	<0.1
ASSuT	681	70	66–73	40	0.19
ACSSuT	319	29	18–43	74	<0.1
ACSuGSTTm	616	27	24–31	—	—

^1^ CI: Confidence interval. ^2^ *I*^2^: Index for the degree of heterogeneity. ^3^ Cochran’s Q-test *p* value.

## Data Availability

All data related to the research are presented in the article.

## Supplementary Materials

The following supporting information can be downloaded at: https://www.mdpi.com/article/10.3390/antibiotics11040532/s1, Table S1: List and characteristics of the 19 eligible studies [13,16,30,34,35,36,40,48,58,62,63,64,65,66,67,68,69,70,71].
