# Supplementary material for: Antibiotic Resistance of Salmonella Typhimurium Monophasic Variant 1,4,[5],12:i:-in China: A Systematic Review and Meta-Analysis
Source: Antibiotics (Basel). 2022 Apr 16;11(4):532. doi: 10.3390/antibiotics11040532 (PMC9031511; doi:10.3390/antibiotics11040532)
Supplement: Supplementary file 1 [file antibiotics-11-00532-s001.zip › antibiotics-1668588-supplementary.pdf]

Table S1. List and characteristics of the 19 eligible studies

References	Language	Year of isolates	Simple sizes	Source	Susceptibility test method
[62]	Chinese	2017-2019	68	Clinical patients	Broth micro dilution method
[63]	Chinese	2008-2019	92	Clinical patients	Broth micro dilution method
[64]	Chinese	2018	124	Foods and clinical patients	Broth micro dilution method
[35]	English	2014-2018	76	Clinical patients	VITEK 2 system
[36]	English	2014-2017	231	Clinical patients	Agar dilution method
[13]	English	2018-2019	28	Animals	Kirby–Bauer disk diffusion method
[65]	Chinese	2016-2017	226	Clinical patients	Broth micro dilution method
[66]	Chinese	2017-2019	143	Clinical patients	Broth micro dilution method
[48]	English	2010-2018	255	Clinical patients and animals	Broth micro dilution method
[16]	English	2011-2016	15	Foods	Kirby–Bauer disk diffusion method
[67]	Chinese	2013-2015	52	Clinical patients	Kirby–Bauer disk diffusion method
[34]	English	2014-2016	56	Animals	Broth micro dilution method
[68]	Chinese	2007-2016	2960	Clinical patients	Kirby–Bauer disk diffusion method
[40]	English	2009-2014	16	Clinical patients	Agar dilution method
[30]	English	2010-2014	13	Clinical patients	Kirby-Bauer disk diffusion method
[69]	English	2007-2012	13	Animals, foods, clinical patients	VITEK 32 GNS-143 card
[58]	English	2011-2014	13	Foods	Kirby-Bauer disk diffusion method
[70]	English	2009-2012	254	Clinical patients	Kirby-Bauer disk diffusion method
[71]	English	2007-2012	1764	Clinical patients	Agar dilution method
